# “With a Little Help from My Friends”: Co-Creating Belonging in Higher Education

**DOI:** 10.3390/bs16020226

**Published:** 2026-02-04

**Authors:** Faiza Aman, Zak Evans, Stephanie White, Arlette Albert, Juliet Foster, Nicola Byrom

**Affiliations:** Department of Psychology, Institute of Psychiatry, Psychology and Neurosciences, King’s College London, London SE5 8AF, UKzak.evans@kcl.ac.uk (Z.E.); juliet.foster@kcl.ac.uk (J.F.)

**Keywords:** student belonging, grassroots initiatives, co-creation, higher education engagement, mixed-methods evaluation

## Abstract

Universities are increasingly seeking ways to build students’ sense of belonging. This paper reports a mixed-methods evaluation of BE At King’s, a seed-funded programme supporting grassroots, co-created initiatives to strengthen connection and inclusion across a large, multi-campus institution. Five projects—ranging from art clubs and community breakfasts to hackathons and writing retreats—were designed and delivered by students and staff, with evaluation embedded from the outset. Quantitative survey data (n = 202) showed high levels of belonging overall, with structured, interactive initiatives most strongly associated with meeting new people and feeling connected. Qualitative thematic analysis highlighted four themes—Refreshing Routines, Inclusive Conditions, Community Leadership, and Layered Engagement—revealing how belonging was fostered through predictable routines, psychologically safe spaces, and opportunities for shared ownership. Bringing findings together shows that grassroots initiatives can engage even less-connected students, but that careful design, inclusive outreach, and sustained facilitation are critical to their success. We argue that universities should embed belonging within the everyday fabric of institutional life through co-produced, flexible, and locally responsive approaches that combine institutional commitment with community leadership.

## 1. Introduction

In the wake of the COVID-19 pandemic, universities renewed their focus on building students’ sense of belonging and connection to campus life. In the UK, this focus was present in reports from the UK Government’s Office for Students (Office for Students, 2022), Advance HE’s What Works programme (Thomas, 2012), and the University Mental Health Charter (Zile et al., 2024). Sector media has repeatedly revisited the challenge of building connection (Dickinson, 2023; Jack, 2022).

The emphasis on belonging reflects a wider concern about students’ absence from campus. Attendance data indicates that many students are spending less time in university spaces (Office for Students, 2022). National surveys report declining motivation and reduced participation (Dickinson, 2023; Neves et al., 2025). Concerns about disengagement are not without nuance. Students may be deliberately managing their time and energy in response to growing financial pressures (Freeman, 2023), caring responsibilities (Spacey et al., 2024), or a preference for more flexible and remote learning environments (Neves & Brown, 2022).

Belonging is a pervasive and powerful driver of behaviour (Baumeister & Leary, 1995), associated with positive education outcomes (Slaten et al., 2016; Thomas, 2012). Belonging is described as an experience of alignment between personal values and social environment (Hagerty & Patusky, 1995), emerging from reciprocity and shared meaning (Mahar et al., 2013). Belonging is underpinned by identification, involvement, and loyalty (Bollen & Hoyle, 1990; Strayhorn, 2018; Tinto, 2012b). Goodenow’s (1993) model of school belonging, centred on feeling respected, included, and supported, has strongly influenced higher education research.

While belonging is frequently highlighted in strategic documents, how institutions foster belonging is less clear. Tokenistic or one-size-fits-all initiatives are often met with student scepticism (Dickinson, 2023). Thomas (2012) observes that retention-focused interventions centred on institutional messaging often have limited impact if they do not address the social and academic dynamics shaping student experience. Building belonging therefore requires more than communications campaigns: it demands trust-building, co-creation, and sustained investment (Masika & Jones, 2016; Thomas, 2012).

Student narratives and practitioner accounts consistently emphasise the relational factors involved in belonging; friendships, mentorship, meaningful connection to staff, and inclusive, participatory learning environments (Maunder, 2018; Thomas, 2012; Wilcox et al., 2005). Grassroots and student-led initiatives may be particularly effective because they are locally grounded, relationship-driven, and responsive to the needs and contexts of the communities they serve (Healey et al., 2014).

Developing an evidence base on the impact and effectiveness of grassroots initiatives is challenging. Evaluation is rarely a priority when such projects first emerge. Many begin as small-scale efforts with small sample sizes, limiting claims of efficacy. To address this gap, we followed grassroots initiatives, seed-funded through the BE At King’s programme and embedded evaluation from the outset.

King’s College London is a large, research-intensive, urban university in the United Kingdom, enrolling over 40,000 students across multiple campuses and disciplines. The student body is diverse. There is a complex organisational structure, with academic departments nested in semi-autonomous faculties, typical of many universities internationally. While the specific institutional context is UK-based, the challenges of fostering belonging, at scale, in large and complex institutions, are likely to be relevant across the sector.

BE At King’s was a research-informed, collaborative initiative designed to foster belonging through locally grounded, community-led projects. Developed in line with the university’s strategic commitment to inclusion, challenge, support, and connection, it supported students, academic staff, and professional services colleagues to co-create initiatives that addressed local belonging challenges. The programme was run by a small team based in the central university services, but rather than relying on top-down directives, the programme adopted a relational, situated, and co-produced approach. Proposals were invited from across the university community and assessed by a joint staff–student panel for clarity, feasibility, potential impact, and opportunities for learning or scalability. Five projects, rooted in lived experience and reflecting the diverse realities of a large, multi-campus institution, were funded and launched in September 2024. These initiatives spanned creative, social, academic and skills-based activities, including a bi-weekly art club, weekly community breakfasts, a PhD writing retreat, a human library, and a hackathon. Together, these initiatives varied in format, duration, and target audience, but were all designed to provide low-barrier opportunities for connection, shared activity, and community building. Here we present a mixed-methods evaluation of these initiatives, focusing on who engages and how participants felt the programmes influenced their sense of belonging. Drawing on learning from the BE At King’s programme, this paper aggregates insights to inform the development of inclusive and connected university cultures.

## 2. General Materials and Methods

### 2.1. Study Design

This study employed a mixed-methods approach to evaluate the impact of grassroots initiatives on participants’ belonging and engagement. Quantitative and qualitative data were collected through pre- and post-engagement surveys. Semi-structured interviews were conducted with initiative participants. The qualitative component was conducted within a critical-realist, experiential framework, emphasising participants’ meaning-making rather than verification of ‘accuracy.’

### 2.2. Participants

For both qualitative and quantitative components of the study, all students and staff who engaged with one or more BE At King’s initiatives were invited to participate. Adverts were presented at all events and where participants signed up in advance, they were emailed an invitation to participate. The study was conducted in accordance with the ethical standards of the institutional research committee and with the Helsinki Declaration and its later amendments. All participants provided informed consent. The study was registered with the institutional ethics review panel: MRA-23/24-45320.

### 2.3. Evaluated Initiatives

Initiatives, summarised in Table 1, are described following the CIDER checklist (Upsher et al., 2025). All initiatives were delivered in-person or in a hybrid format.

### 2.4. Study 1: Quantitative Data

Quantitative data was used to understand the profile of individuals participating in initiatives and to assess whether there were any differences across initiatives in their impact on belonging.

#### 2.4.1. Procedure

Participants were invited to complete a short online survey both before (Time 1) and after (Time 2) engaging in the initiative. Demographic data were collected in the first survey. Participants had the option to answer demographic questions or provide their university ID for key characteristics to be drawn from the university record (i.e., sex, age, faculty, level of study, home/international status, staff pay grade).

The survey incorporated items to assess belonging and engagement. Items were taken from an existing university-wide evaluation of student wellbeing and the UBelong longitudinal survey. In this way, the items used had been previously tested with university students. To maximise participation, surveys were as short and simple. Questions and process for analysis are summarised in Table 2.

#### 2.4.2. Data Analysis

The engagement score was used to classify participants as Less Engaged (<3.5) or Highly Engaged (>3.5). Welch’s ANOVA was used to test for differences in mean belonging scores between projects. Unlike the traditional one-way ANOVA, Welch’s ANOVA does not assume equal variances and is more robust when group sizes are unequal, both of which applied in our data (Delacre et al., 2017; Welch, 1951).

### 2.5. Study 2: Qualitative Data

Interviews were used to better understand the experience of individuals running initiative and engaging with the initiatives.

#### 2.5.1. Participants

In total, 13 participants took part in interviews, including 6 participants as well as 7 project leaders who developed and facilitated these activities. Demographics for interview participants are summarised in Table 3. Data was anonymizsed prior to analysis to protect confidentiality.

#### 2.5.2. Procedure

Semi-structured interviews, conducted online, lasted between 30 and 45 min. These were audio-recorded with consent and transcribed verbatim. Interviews addressed: (1) motivations for joining the initiative (e.g., How did you find out about…?; What motivated you to participate in…?); (2) initial expectations and perceived value of participation (e.g., What did you hope to gain from attending…? What new did you learn at…?); (3) changes in sense of belonging, wellbeing, or engagement (e.g., How did participating in … affect your sense of belonging? How effectively did … provide a space to meet new people?); (4) suggestions for future improvements.

#### 2.5.3. Data Analysis

Interview transcripts were analysed using inductive thematic analysis (Braun & Clarke, 2006), and our process was checked against the reporting guidelines (Braun & Clarke, 2025). The analysis focused on identifying project impact on sense of belonging and connection, as well as motivators and barriers to engagement. Two researchers (FI & AA) independently coded the data, before discussing codes to deepen interpretation. Themes were developed through an iterative process, supported by regular discussion with a third researcher (NB). The analysis team included research assistants, students, and an established researcher with extensive experience teaching in higher education.

## 3. Results

### 3.1. Quantitative Data

A total of 792 staff and students, aged 18 to 66+ years old, were recorded as participating in the five initiatives, of which 202 responded to the survey. Table 4 outlines the demographic characteristics of participants. More women engaged with initiatives than men and graduate students were more likely to engage than undergraduates. These patterns are present even when looking at attendance, rather than participation in the evaluation. Attendance data for the Community Breakfast showed that staff engaging were more likely to be in the lower university pay grades. In total, 9 individuals completed an evaluation for the Hackathon. Due to the small sample size demographic details could not be extracted from the university record.

**Figure 1:**
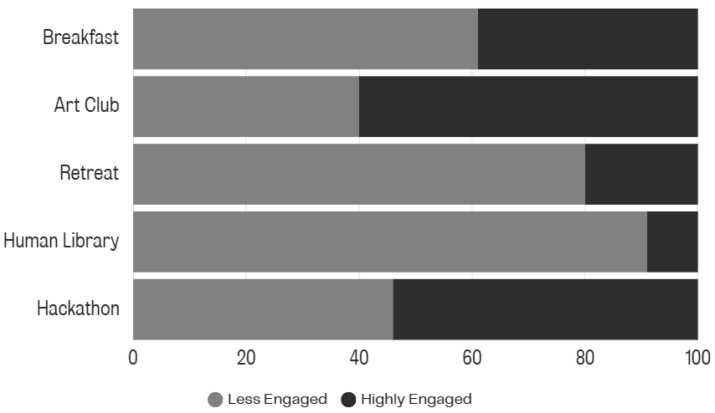
Proportion of participants identified as ‘Less Engaged’ across initiatives.

As shown in Figure 1, across all projects, 40% of the participants were classified as ‘Less Engaged with University Life’ based on their Time 1 data. This provides a profile of who was likely to engage with initiatives. The Human Library and the Writing Retreat were successful in connecting with individuals who did not feel they were well engaged with wider university life.

Participant sense of belonging, after the initiatives (Time 2), is summarised in Table 5. Belonging scores were comparable across initiatives for “I feel like I belong,” F (4, 196), = 1.93, *p* = 0.17, and “I feel like I fit in,” F (3, 187) = 1.13, *p* = 0.34. There was significant variation across the initiatives for “I feel more connected to the university,” F (3, 182) = 9.63, *p* < 0.001. Here, participants in the Hackathon showed higher levels of connection after the initiative than participants in all other initiatives, except for the Art Club. There was also significant variation across initiatives for “I met new people,” F (2, 174) = 49.98, *p* < 0.001. While participants of the Art Club and Hackathon agreed that they met new people, this was significantly less for participants at the Breakfasts.

### 3.2. Qualitative Data

**Figure 2:**
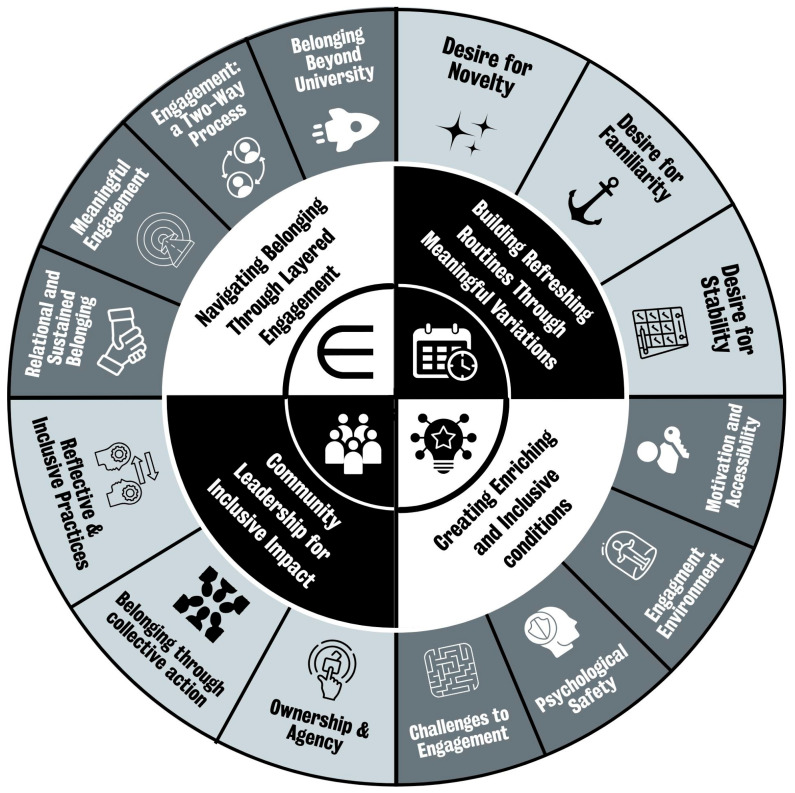
Summary of themes from qualitative analysis.

The analysis generated four interrelated themes, summarised in Figure 2, that illuminate the interplay between individual agency, collective leadership, and institutional design. Refreshing Routines highlights the need to balance stability with change to sustain wellbeing and interest, while Inclusive Conditions emphasises designing activities that are accessible, empowering, and conducive to meaningful engagement. Community Leadership and Layered Engagement together demonstrate how co-creation, collective action, and institutional support enable belonging to unfold within and beyond the university over time, underscoring the importance of flexible, co-created, and responsive engagement strategies.

#### 3.2.1. Theme 1: Building Refreshing Routines Through Meaningful Variations

Participants sought to balance the predictability of routine with the stimulation of change. Many described wanting a break from the monotony of daily work and study, to re-energise, explore new interests, or experience something different. Novelty was actively sought to maintain engagement and prevent burnout.

“*My life is I wake up when I go to the library and then I leave the library and then I go home and then I sleep. Unfortunately*.”

(CB1)

The desire for novelty extends to meeting new people, to be “*part of a community to get to know students, other students and staff,*” (AC2) and explore new spaces, “*I was glad to have a space other than daily office*” (CB2).

Novelty did not always mean a completely activity. Familiarity could provide comfort, motivation, and a sense of identity, particularly during times of transition or stress. Several participants described returning to past hobbies to re-establish normalcy; “*Let me try something that I used to really enjoy... I had it in my undergrad... I’d always go there with my friends after training*” (CB1). Activities that mirrored past experiences were seen as approachable and emotionally resonant, helping participants feel grounded. These acted as anchors in unfamiliar settings, encouraging continued engagement.

Alongside novelty, participants valued stability and consistency, seeking to integrate new experiences into weekly routines to create rhythm and predictability: “*I have something to look forward to on Tuesdays. I’m going to plan my day around it*” (AC1), “*If I have it in my routine, I will continue to do it*” (CB1). Indeed, for relationship building the consistency could be crucial, “*It’s not a one-time thing. That’s what I really enjoy… I can keep on meeting the same people that I’ve already met*” (AC1). Participants also appreciated opportunities for small variations within routines.

Some participants were looking for drop-in flexibility, as work schedules made it hard to commit weekly: “*Sometimes I can’t go because my experiments are running … I can’t just leave*” (AC1). For these participants, the ability to attend when possible was crucial.

Engagement activities were viewed as opportunities to de-stress and prioritise wellbeing, with both novelty and predictability seen as essential. Overall, participants sought a blend of routine and variation that supported energy, comfort, and ongoing engagement.

#### 3.2.2. Theme 2: Creating Enriching and Inclusive Conditions

Inclusive and empowering activities are shaped not only by what is offered but by how, where, and for whom they are delivered. Creating the right conditions for engagement requires addressing barriers, enabling access, and fostering environments where people feel safe, seen, and motivated to participate. One of the most persistent challenges participants identified was “*spreading awareness in big university contexts*” (BN).

Even well-designed activities risk being lost in the noise. Without clear, targeted communication, opportunities could easily go unnoticed. And even when awareness was achieved, sign-up did not always lead to attendance; “*students... would just book the ticket without showing up*” (WR). Further, the timing of events can be challenging: “*Most activities … are not the times that I would usually do activities—I would usually have either dinner or I would work*” (AC1). While meeting in person was valued by many, others felt that more institutional effort to connect with remote students was essential: “*I am away from the campus … [is] King’s considering me even if I am away? … I would like more online support*” (CB2).

To counter these challenges, participants emphasised the need for diverse opportunities that reflect the varied interests of the student body. Groups such as PhD students or those based on less central campuses felt overlooked: “*I don’t think there are a lot of activities for PhD students… everything is based around the undergraduate experience*” (AC1).

Once engaged, participants stressed that ease of access was key. Activities that were simple to join and required minimal preparation were especially welcoming:

“*I would say that everything is provided for, so I didn’t have to think about, oh, I need to buy a canvas… because I don’t have any background with it*”

(AC1)

“*I just like the fact that I could just show up… I didn’t have to sign up for anything*”

(CB1)

Participants and project leaders across a range of initiatives valued activities that were guided but flexible, providing enough structure to avoid confusion while leaving room for personal expression:

“*I think people know that they can do their own thing, but like [the project organiser] is there for questions…*”

(AC2)

“*Somebody is keeping time… but you are free to just get up if you want to stretch*”

(WR)

“*I was pleased to see that there were a variety of books… so you could just pick whichever was most relevant to you*”

(HL1)

The physical and psychological atmosphere also shaped engagement. A calm, welcoming setting with music, friendly hosts, and a sense of routine fostered connection: “*Like the sense of community, like the chill vibes… the music in the background, [project leader] being super friendly and hosting us…*” (AC1). Conversely, unexpected noise or unfamiliar people could feel disruptive: “*It gets very loud and I could tell that the other [attendees]… were like a bit like put off*” (CB1).

Psychological safety was essential. For some, taking the first step was challenging. Some felt that they needed the support of a friend to make this first step: “*It was like a safe environment, but the first time I did go with somebody from my [team]… but now I just attend them by myself*” (AC1). Effective facilitation at the event was crucial to a positive experience: “*I was quite nervous when I first went … but [the organiser] guided me to try watercolour … I really enjoyed that*” (AC3).

Ultimately, participants were drawn to opportunities that allowed them to de-stress and recharge, offering a counterbalance to academic and personal demands: “*My main factor was really to do… a bit of art that I thought was fun and in like a chill setting… nothing to worry about*” (AC2). Tangible outcomes—such as taking home artwork, receiving certificates, or seeing visible change—reinforced motivation and even inspired others to join: “*I took [my artwork] to my office and everybody… really enjoyed it… and afterwards I motivated two other people to join as well*” (AC1).

Together, these reflections show that creating enriching and inclusive conditions requires more than just offering activities. It involves intentional design, inclusive outreach, and supportive environments that meet people where they are. When these elements combine, engagement becomes more accessible, meaningful, and sustaining.

#### 3.2.3. Theme 3: Community Leadership for Inclusive Impact

Participants, especially those leading projects, emphasised the importance of autonomy and the ability to make a difference. Being supported to lead their own ideas, whether by planning events, proposing initiatives, or shaping project direction, strengthened their sense of connection and commitment. Leaders often spoke of projects as their own, “our hackathon,” “our project plan,” signalling identification with their ideas and a motivation to see them succeed:

“*I think it was a wakeup call for how unprepared [for employment after university] you can feel sometimes... And then we thought, but we can do something about it then; I’m happy to come up with a proposal, speak to my department about it...*”

(BN)

However, project leads also reflected on resource challenges: “*We don’t have any departmental budget*” (CB). A student organising a project noted that they had organised their whole budget around catering without realising that the university would charge them for using microphones and AV support, meaning they “*had to redo everything… it was frustrating, it was just very unexpected*” (HK). They highlighted the need for more “*help planning in the long term*” (HK) as well as institutional support and shared organising responsibilities to sustain initiatives without overburdening individuals. For the community breakfast, despite efforts to encourage other departments to engage with the breakfast, so that they could offer their event attendees a free breakfast, there was “*little success*” (CB) engaging other teams across the university.

A sense of ownership was reinforced by tangible outcomes and visible progress, with participants eager to see that their efforts contributed to a broader, purposeful journey: “*How can we show that we’re working towards that right? What’s the action plan to achieving the purpose after the actual meeting?*” (HL1). Organising itself became a form of participation, paving the way for collective action and a deeper sense of belonging. Individuals participating in initiatives stressed that co-organising fostered community: “*I think it it’s probably better to have more than one person organising this kind of activities cause organising the activity is also a way to have this kind of sense of belonging*” (WR). Involving beneficiaries in planning and delivery, even in small ways such as logistics, created shared responsibility and opportunities to form new relationships through shared goals: “*I really made some new friends... organising this kind of activity*” (WR).

This collaborative approach also enabled more inclusive and reflective practices. Including both organisers and beneficiaries enriched understanding of needs, while diversity in leadership ensured initiatives were representative and responsive: “*I think it’s a really good... bottom-up approach to it and actually... it has the greatest potential to meet the needs*” (BN). Participants described how grassroots leadership challenged assumptions about who engages and where, noting unexpected enthusiasm from campuses thought to be disengaged:

“*The campus that is one of the busiest was unexpected. So that’s definitely surprising because it shows that... some of the less-engaged campuses can be really engaged with the right thing.*”

(CB)

These reflections highlighted that while communities are diverse, many needs—such as connection, recognition, and purpose—are widely shared. Creating space for dialogue and experimentation allowed participants to design initiatives that were both tailored and inclusive. Organising was seen not as the responsibility of a few but as an open invitation to participate, with the act of organising itself becoming a catalyst for deeper involvement. In this way, community leadership functioned less as top-down direction and more as shared ownership, enabling participation, reflection, and more resilient forms of engagement.

#### 3.2.4. Theme 4: Navigating Belonging Through Layered Engagement

Participants described belonging as a gradual, relational process that evolves over time, shaped by meaningful, reciprocal interactions and personally relevant experiences that often extend beyond the university. It was often anchored in specific groups or teams and nurtured through regular, ongoing activities that built familiarity and trust. Consistent touchpoints helped transform casual encounters into friendships:

“*It’s not a one-time thing. That’s what I really enjoy… I can keep on meeting the same people that I’ve already met.*”

(AC1)

“*I met [the project leader]. So we’re friends now… So we’ve met outside of the Art Club.*”

(AC2)

Importantly though, belonging was not achieved through all projects, or for all participants. One participant of the art club noted that “*I wasn’t really talking to a lot of people … I was so focused on painting*” (AC3). Another participant who’d attended the community breakfast didn’t feel that this was designed to encourage connection, the “*Atmosphere was not something like to actively meet new people …*” (CB2). They remind us that “*conversations don’t happen without any triggers*” (CB2).

Belonging was experienced as contextual and relational, often rooted in smaller communities rather than the whole institution. For many participants, their sense of connection was tied to a particular department, peer group, or shared interest, rather than to the wider university: “*[Belonging] is more like my office and department based, but not really King’s based*” (AC1).

Engagement was seen as essential to building this kind of belonging and was described as a two-way process involving both institutional and individual responsibility. While institutions were expected to provide diverse opportunities to engage, participants also saw themselves as responsible for choosing how and when to participate. They valued autonomy in ways suited to their interests, energy levels, and social preferences, alongside gentle structure and facilitation that encouraged interaction without pressure:

“*I think people know that they can do their own thing, but she’s there for questions and I have seen students…ask her information about how to do water colouring techniques….*”

(AC2)

“*There must be some actively and explicit reason nudging participants to connect and talk to others.*”

(CB2)

Participants also stressed the importance of meaningful engagement, where quality of experience mattered more than the number of attendees. Smaller, more intimate settings were often preferred, as they enabled deeper conversations and a more comfortable atmosphere. Some participants even described feeling connected simply by sharing space with others:

“*I wasn’t really talking to a lot of people, but it is quite nice to kind of hear other conversations.*”

(AC3)

“*I would say there’s not a lot of people… every session approximately has like 10 people… you get to like chat with each other without it being too crowded.*”

(AC1)

Engagement needed to feel purposeful and worthwhile to be sustained. Participants appreciated activities that added value to their personal or professional lives, motivating them to return: “*I was pleased to see that… that really made it worthwhile and directly relevant to my job*” (HL1).

Some activities were more effective at creating engagement than others. For some participants, engagement was more functional than relational, valued as a way to access free food, change routines, or increase productivity: ‘I didn’t meet new people … that’s just me.’ For these individuals, belonging was experienced more as a quiet co-presence than as active community-building.

Finally, participants noted that belonging and engagement were not confined to the university. Their sense of connection often extended beyond campus, through friendships, local communities, and personal projects, which were seen as equally important:

“*I really like this sound of projects and things like that and doing things outside of my degree. I believe that’s where I thrive and that’s where a lot of personal growth is experienced for me*”

(HK1)

These reflections suggest that belonging cannot be engineered through one-off events or one-size-fits-all initiatives. Instead, it is built through layered, context-sensitive engagement that supports individuals to connect in ways that resonate with them. This requires institutions to design inclusive, flexible, and responsive opportunities. When institutions and individuals contribute together, belonging becomes sustainable, authentic, and personally meaningful.

## 4. Discussion

This evaluation of a grassroots initiative to foster belonging highlights the central role of relationships and the positive impact of co-creation on agency and autonomy. Together, the quantitative and qualitative findings converge to show that grassroots initiatives can meaningfully strengthen belonging when they are relationally grounded, co-produced, and designed to foster repeated, meaningful encounters. Structured, interactive activities were most strongly associated with connection, and qualitative data explain why: participants valued psychologically safe, well-facilitated spaces that encouraged interaction. Importantly, the participation of less-engaged students demonstrates that low-barrier, authentic opportunities can extend the reach of engagement efforts.

Quantitative data analysis indicates that both staff and students engaged with the initiatives, though undergraduate engagement was weaker. Around 40% of participants were classified as ‘less engaged,’ with the Human Library and Writing Retreat attracting particularly high proportions of these individuals. This suggests that well-designed grassroots initiatives can reach those marginalised or disconnected from mainstream university life (Masika & Jones, 2016; Maunder, 2018).

Belonging scores were generally high, but people participating in structured, interactive initiatives such as the Hackathon and Art Club were particularly likely to report that the initiative helped them meet new people. This finding supports evidence that intentionally designed, interactive formats are most successful at fostering peer connection and strengthening institutional attachment (Thomas, 2012; Wilcox et al., 2005). In contrast, routine or transactional activities like the Community Breakfasts, though positively received, created fewer opportunities for new social ties. This underscores the need to move beyond passive provision of space towards activities that actively cultivate interaction (Dickinson, 2023; Healey et al., 2014).

Qualitative analysis identified four interrelated themes, Refreshing Routines, Inclusive Conditions, Community Leadership, and Layered Engagement, that reaffirm belonging as a dynamic, relational process (Bollen & Hoyle, 1990; Covarrubias, 2024; Slaten et al., 2016). Contemporary scholarship emphasises that belonging in higher education is not a single feeling but a multidimensional experience shaped by relationships, place, and institutional culture (Ahn & Davis, 2020; Allen et al., 2024; Antonsich, 2010). Our themes echo this: structured, interactive initiatives were most strongly associated with meeting new people and feeling connected, consistent with work highlighting the centrality of facilitated social connection for engagement and institutional attachment (Thomas, 2012). At the same time, the qualitative themes demonstrate that connection is enabled (or constrained) by conditions of access, safety, and recognition—suggesting that “opportunities to connect” are necessary but insufficient without inclusive design.

Participants valued opportunities to take ownership and co-create initiatives, aligning with research emphasising that belonging is cultivated through interaction, reciprocity, and shared meaning rather than top-down messaging (Baumeister & Leary, 1995; Covarrubias, 2024; Mahar et al., 2013; Thomas, 2012). Emerging evidence positions co-creation and student–staff partnership as central mechanisms for fostering belonging and inclusion. Studies from De Montfort University, the University of Brighton, and more recent work by Shakir (2024) demonstrate that participatory, dialogic, and partnership-based approaches can strengthen students’ sense of connection to their institution, particularly for groups historically marginalised within higher education (Ansley & Hall, 2019; Hall et al., 2022; Shakir, 2024). Co-creation operates through shared decision making and collaborative design (Dollinger & Lodge, 2020) and can strengthen students’ sense of agency, emotional connection, and belonging (Wilson et al., 2024).

Grassroots approaches further challenge assumptions about who engages, broaden the range of voices shaping initiatives, and allow organisers to draw on their lived experience and networks to design more inclusive activities. This is particularly important given ongoing debates about whether belonging implies integration into existing norms or identity-affirming inclusion within majority-led institutions. Recent syntheses caution that belonging risks becoming conceptually entangled with “integration,” and that uncritical use can reproduce assimilation pressures (Covarrubias, 2024; Tinto, 2012a). The processes described by participants map more strongly onto recognition and inclusion, psychological safety (ease and security), perceived care and support, and relational cohesion than to conformity-based “fitting in” (Allen et al., 2024; Dias-Broens et al., 2024). Grassroots, co-created initiatives may be especially well suited to this framing because they allow belonging to be locally negotiated and enacted through shared ownership, reciprocity, and trust.

Engagement was seen as a shared responsibility: participants wanted autonomy to choose how to engage but also appreciated gentle structure to support participation. Belonging was experienced as layered and context-specific, anchored in repeated, meaningful encounters, supported by psychologically safe environments (Goodenow, 1993; Maunder, 2018) and often rooted in smaller peer groups or departments rather than the whole university (Stachl & Baranger, 2020). Giving students control and choice boosts agency and contributes to engagement and belonging (Brandt, 2024). Agency, competence, and relatedness can foster belonging and wellbeing (Ryan & Deci, 2000). Initiatives may be most successful when nested in micro-communities.

Initiatives that combined routine with novelty were especially valued, offering predictable, restorative pauses alongside opportunities for new experiences. This aligns with evidence that routines support wellbeing and reduce burnout, while novelty sustains motivation (Puranitee et al., 2022; Romeo et al., 2024). In this way, grassroots initiatives acted not only as spaces for connection but also as mechanisms for maintaining energy and emotional balance across the term.

These findings reinforce longstanding critiques of top-down or performative belonging strategies (Covarrubias, 2024). Students are quick to identify when initiatives feel tokenistic or disconnected from their lived experiences (Dickinson, 2023; Jack, 2022). The appeal of BE At King’s appears rooted in its co-productive ethos and low-barrier design, which enabled participants to shape and own the interventions they joined. This supports calls for institutions to move away from one-size-fits-all approaches and towards diverse, relationally grounded practices of inclusion (Masika & Jones, 2016).

### 4.1. Limitations and Future Directions

These findings underscore ongoing challenges in evidencing the impact of small-scale grassroots work. Variation in sample size and design across projects limits the generalisability of conclusions, and the short timeframe for evaluation constrains our ability to assess sustained outcomes. Women and postgraduate students were more likely to engage, highlighting persistent equity issues. While the study captures variation in engagement across roles and levels of study, it was not designed to examine intersectional differences in belonging (e.g., across race, gender, disability, socio-economic background, or caring status), limiting our ability to understand how experiences of co-created initiatives may differ for students and staff positioned differently within institutional power structures. Participants reflected on the challenge of spreading awareness and securing attendance. Simply supporting grassroots initiatives operationally may be insufficient without robust and inclusive outreach strategies. Future research should explore ways to embed belonging initiatives into institutional strategy while preserving their grassroots character. The sector needs evaluation approaches that are proportionate yet capable of capturing long-term change (Dias-Broens et al., 2024; Priestley et al., under review).

### 4.2. Implications for Practice

Findings from this evaluation offer practical insights for supporting belonging through grassroots, community-led engagement. Drawing on the BE At King’s programme, we propose a flexible “Belonging-by-Design” model with four phases that balance institutional strategy with local ownership. This model is grounded in core principles that ensure relevance, inclusivity, and impact. Belonging is cultivated through authentic interaction rather than passive messaging, highlighting the need to prioritise opportunities for genuine engagement. Diverse initiatives are essential to reach and resonate with different groups across the university community. Relationships, both informal and structured, play a central role in creating supportive environments. Shared leadership empowers students and staff, fostering ownership and long-term sustainability of efforts. Finally, meaningful evaluation should capture not only outputs but also the nuanced lived experiences of participants, using both quantitative and qualitative methods.

#### 4.2.1. Foundations—Creating the Conditions

The success of grassroots initiatives depends on a clear institutional commitment to inclusion, wellbeing, and connection. Providing funding, resources, and light-touch administrative support empowers grassroots organisers, while visible senior endorsement signals their importance. Open, accessible opportunities for students and staff to submit proposals are essential to encourage broad participation.

#### 4.2.2. Co-Creation—Designing with Communities

Meaningful initiative design centres on empowering community members to shape activities that reflect their lived experiences. Institutions should encourage a wide range of project types, including creative, academic, social, or identity-based. Offering mentoring and planning support can help leaders refine ideas and build capacity. Belonging should be framed as a continuous process, with inclusive principles embedded throughout design and delivery.

#### 4.2.3. Delivery—Implementing Responsively

Initiatives should be implemented in a responsive, inclusive, and context-sensitive way, keeping them low-barrier and accessible. Institutions should offer flexibility in delivery style and location, including multi-campus options, and prioritise relational spaces over formal event structures to foster genuine connection. Practical institutional support must be balanced with local autonomy to maintain community ownership.

#### 4.2.4. Reflection—Capturing Impact

Evaluating grassroots initiatives is essential for their sustainability and growth. Mixed-methods approaches, combining surveys and interviews, as well as both validated and custom measures of engagement, belonging, wellbeing, and satisfaction, provide a comprehensive understanding of outcomes. Including project leader reflections alongside participant feedback offers a fuller picture of impact, and sharing insights across the institution and sector helps inform and strengthen future initiatives.

## 5. Conclusions

These findings reinforce calls for universities to embed belonging within the everyday fabric of institutional life through co-produced, flexible, and context-sensitive approaches (Healey et al., 2014; Masika & Jones, 2016). This evaluation demonstrates that grassroots, co-created initiatives can make a meaningful contribution to students’ sense of belonging when they are low-barrier, relationally rich, and responsive to local contexts. The convergence of quantitative and qualitative findings shows that structured, interactive activities are particularly effective at fostering connection. Intervention success depends on predictable routines, inclusive conditions, and opportunities for shared ownership. Belonging was experienced as layered and context-dependent, rooted in smaller communities and sustained through repeated, meaningful encounters. Together, these insights argue for an approach that combines institutional commitment with local leadership, moving beyond top-down messaging to create authentic, co-produced opportunities for engagement. There are practical challenges around awareness-raising, sustaining participation, and evaluating emergent initiatives, that must be addressed if belonging is to become a sustained, equitable feature of university life, enabling all students to feel connected, supported, and able to thrive.

## Figures and Tables

**Table 1:** Details of the Be At King’s funded projects.

Name & Description	Purpose	Audience	Project Lead	When?	Where?	Duration
**The Art Club**: an inclusive space where students and staff can come together to socialise and relax while creating art.	To (i) increase a sense of belonging and community and (ii) generate greater knowledge about the positive impact of art on mental health and wellbeing.	All staff and students	Faculty Administrative Coordinator	Every two weeks	University Sports and Wellness Centre	90 min
**Community Breakfast**: a space for the whole community to come together and meet new people over a free morning meal.	To (i) encourage staff and students to meet new people and socialise through an eat-in-only policy and (ii) provide support to those who might struggle financially by providing healthy food for free.	All staff and students	University Catering Team	Every week	Canteens across campus	2 h
**PhD Writing Retreat**: An opportunity for PhD and postgraduate students to enjoy two days amongst nature, during which they would engage in structured writing sessions.	To help students develop methods to improve concentration and be productive to support their future studies. Students were encouraged to support and talk to one another, sharing their experiences and building their networks.	Postgraduate taught and research students	PhD Student	A one-off event	Lee Valley Regional Park	2 days
**Human Library**: A safe, non-judgmental space for staff members to ask questions and share experiences that they would not usually get a chance to talk about.	To open dialogue about personal experiences, expertise, cultures, and identities; to promote empathy and connectivity within the diverse King’s community.	Staff members	Programme Manager in Organisational Development	2 events	Hybrid	2 h
**Fast Fashion Hackathon**: A team-based ideation competition where students engage with skill-building workshops and industry talks, as well as build a pitch as a group addressing fast fashion and environmental concerns.	To provide (i) an opportunity for students to meet other students, (ii) a space for students to build employable skills, and (iii) encourage innovative ideas responding to pressing global issues.	Undergraduate Students	Two Undergraduate students	A one-off event	On campus	2 days

**Table 2:** Survey questions.

Domain (Response Scale)	Questions	Processing
Engagement (5-point scale; 1—never, 2—once or twice a year, 3—monthly, 4—weekly, and 5—daily)	How often do you engage with: (1) in-person activities on campus. (2) socialising with your university. peers/colleagues outside of an organised group. (3) making use of university spaces to spend time by yourself.	Engagement score = average score to three engagement questions.
Belonging (5-point Likert scale; 1—Strongly disagree to 5—Strongly agree)	To what extent do you agree with the following statements: (1) I feel like I belong. (2) I met new people at [initiative name]. (3) Participating in [initiative name] has made me feel more connected to the university community. (4) I feel like I fit in.	Each item analysed individually.

**Table 3:** Interview participant demographics.

Participant	Sex	Role in BE at King’s	Role in University	Project
AC3	Female	Participant	PhD Student	Art Club
AC1	Female	Participant	PhD Student	Art Club
AC2	Female	Participant	Staff	Art Club
CB2	Female	Participant	PhD Student	Breakfast
CB1	Female	Participant	Master’s Student	Breakfast
HL1	Female	Participant	Professional Services Staff	Human Library
ACPL	Female	Project Lead	Professional Service Staff	Art Club
CBPL	Female	Project Lead	Professional Service Staff	Breakfast
HKTPL1	Female	Project Lead	Undergraduate Student	Hackathon
HKTPL2	Female	Project Lead	Undergraduate Student	Hackathon
HLPL	Female	Project Lead	Professional Service Staff	Human Library
WRPL	Male	Project Lead	PhD Student	Writing Retreat

**Table 4:** Participant demographics by projects.

	Art Club (Survey)		Community Breakfast Attendees		Community Breakfast (Survey)		Writing Retreat (Survey)		Human Library (Survey)	
	N	% of Total	N	% of Total	N	% of Total	N	% of Total	N	% of Total
Total	30		681		139		10		14	
Male	2	7	243	36			3	30	1	7
Female	17	57	403	59			7	70	6	43
Students	23	77	401	59	76	55	10	100	0	0
1st year UG	9	30	84	21	8	11	0	0		
2nd Year + UG	2	7	93	23	16	21	0	0		
PGT	2	7	167	42	31	41	1	10		
PGR	10	33	53	13	18	24	9	90		
Staff	4	13	280	41	52	37	0	0	14	100
Academic	0	0			7	5			4	29
Researcher	1	3			8	6			1	7
Professional Services	3	10			30	22			4	29

**Table 5:** Descriptives of participants scores on questions measuring sense of belonging across the different projects.

Projects	I Feel Like I Belong		I Feel More Connected to the University Community		I Met New People		I Feel Like I Fit In	
	X (SD)	N	X (SD)	N	X (SD)	N	X (SD)	N
The Art Club	4.30 (0.95)	30	4.47 (0.68)	30	4.17 (1.09)	30	4.27 (0.74)	30
Breakfasts	4.00 (1.11)	138	3.99 (1.06)	138	2.91 (1.36)	139	3.99 (1.01)	138
Hackathon	4.33 (0.50)	9	4.50 (0.53)	8	4.88 (0.35)	8	4.11 (0.93)	9
Human Library	3.71 (1.49)	14					3.64 (1.39)	14
Writing Retreat	4.10 (0.57)	10	3.90 (0.57)	10				
Overall	4.04 (1.09)	201	4.09 (0.99)	186	3.21 (1.42)	177	4.02 (0.97)	191

## Data Availability

Data is available upon reasonable request to corresponding author.
